# Multifocal Epithelial Hyperplasia of Oral Cavity Expressing HPV 16 Gene: A Rare Entity

**DOI:** 10.1155/2013/871306

**Published:** 2013-12-24

**Authors:** M. P. V. Prabhat, Chintamaneni Raja Lakshmi, N. Sai Madhavi, Sujana Mulk Bhavana, Gummadapu Sarat, Kodali Ramamohan

**Affiliations:** ^1^Department of Oral Medicine and Radiology, Drs. Sudha and Nageswara Rao Siddhartha Institute of Dental Sciences, Gannavaram Mandal, Krishna District, Andhra Pradesh 521286, India; ^2^Department of Oral and Maxillofacial Surgery, Drs. Sudha and Nageswara Rao Siddhartha Institute of Dental Sciences, Gannavaram Mandal, Krishna District, Andhra Pradesh 521286, India

## Abstract

Focal epithelial hyperplasia is a rare contagious disease caused by human papilloma virus. Usually HPV involves either cutaneous or mucosal surfaces, whereas concomitant mucocutaneous involvement is extremely rare. We report such a unique case of multifocal epithelial hyperplasia involving multiple sites of oral cavity along with skin lesions in a 65-year-old female. We also discuss the probable multifactorial etiology and variable clinical presentations of the lesions, including evidence of HPV 16 expression, as detected by polymerase chain reaction. The present report illustrates the need for careful examination and prompt diagnosis of the disease, as it might be associated with high risk genotypes such as HPV 16 and 18.

## 1. Introduction

Human papilloma virus (HPV) infection is known to induce proliferative lesions on the skin and mucosa which may be benign or malignant. Over 120 genotypes have been identified in the HPV family, among which HPV 16 and 18 are considered to be high risk genotypes owing to their malignant potential. Benign oral lesions associated with HPV include squamous papilloma, verruca vulgaris, condyloma acuminatum, focal epithelial hyperplasia, and malignant variants like verrucous carcinoma and squamous cell carcinoma [1].

Focal epithelial hyperplasia of the oral cavity is a benign infectious disease caused by HPV, clinically presenting as multiple, well circumscribed papules on the oral mucosa, primarily involving gingiva, buccal, or labial mucosa [2]. The lack of sufficient literature and the asymptomatic nature of the condition make the clinical diagnosis difficult.

We hereby report a unique case that presented clinically as multifocal epithelial hyperplasia of the oral mucosa in concurrence with the skin lesions.

## 2. Case Report

**Figure 1:**
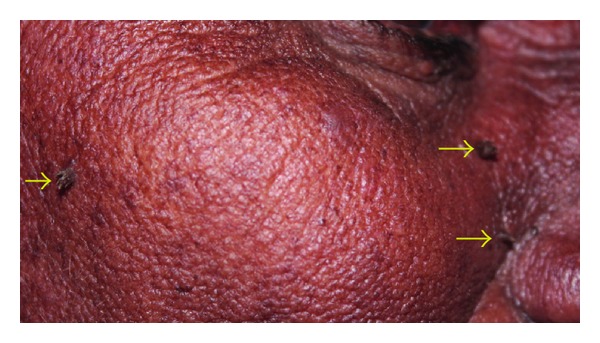
Multiple warts were seen involving face.

**Figure 2:**
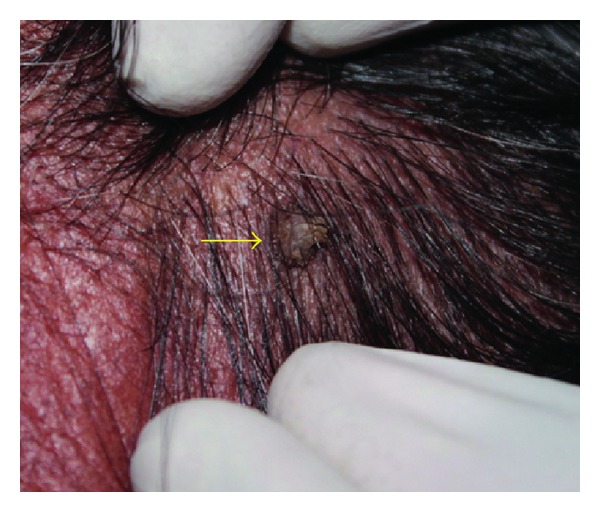
Wart seen on the scalp.

**Figure 3:**
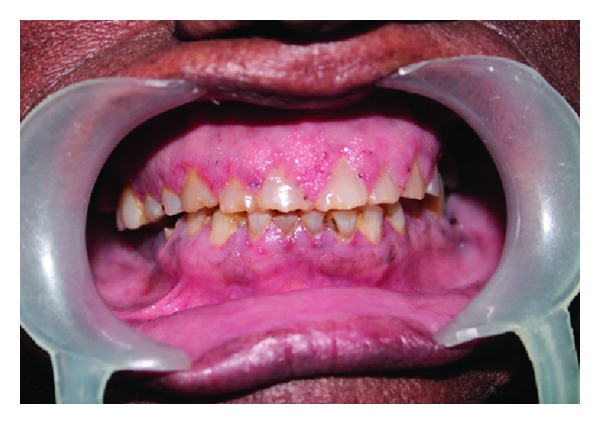
Multiple coalescent papillary projections involving upper and lower anterior gingiva.

**Figure 4:**
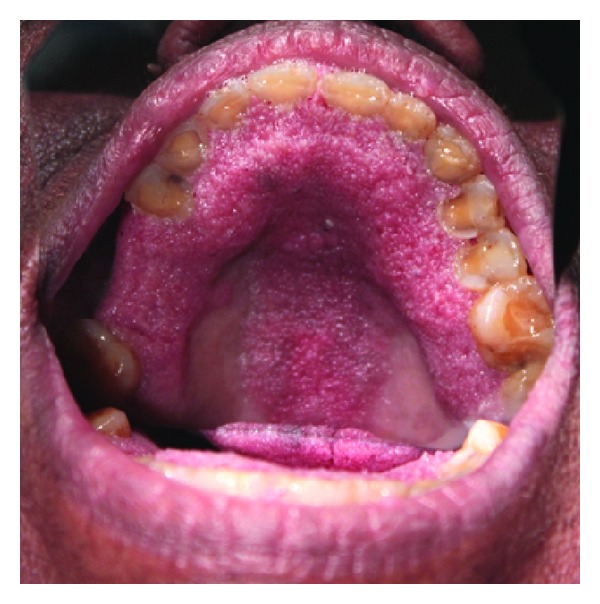
Extensive lesion seen involving palate.

**Figure 5:**
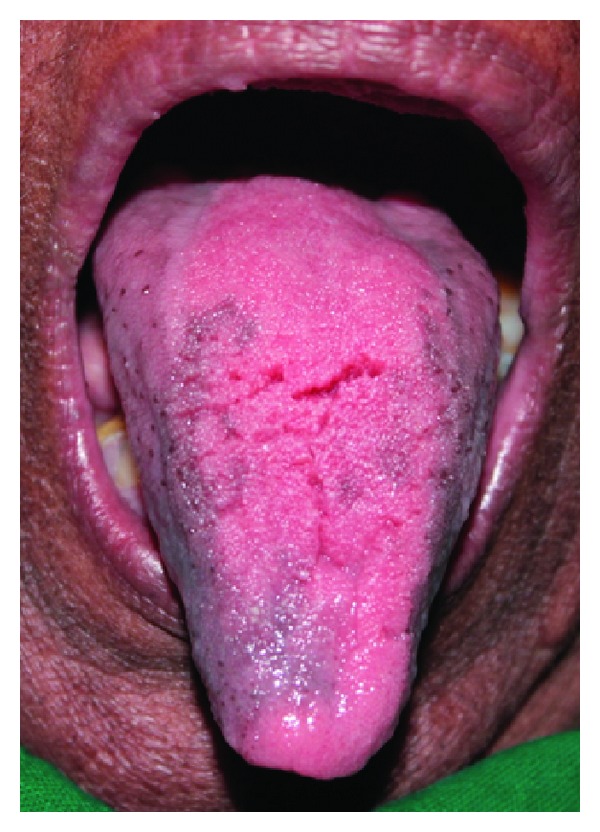
Papillary lesions with fissuring seen on the dorsum of tongue.

**Figure 6:**
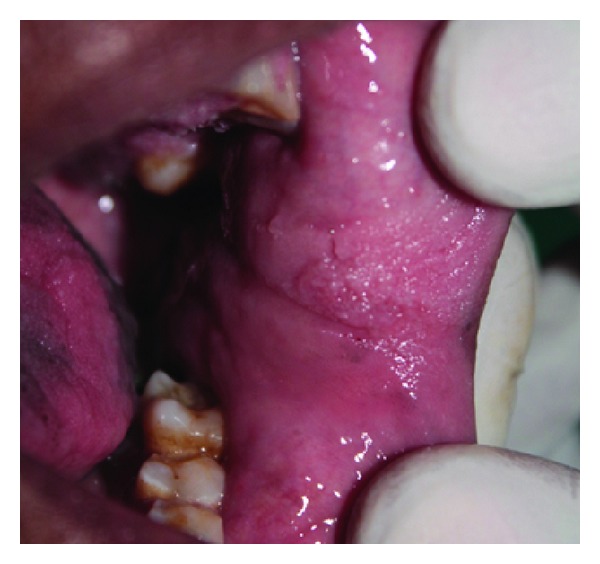
Papillary lesion at the left commissural area.

**Figure 7:**
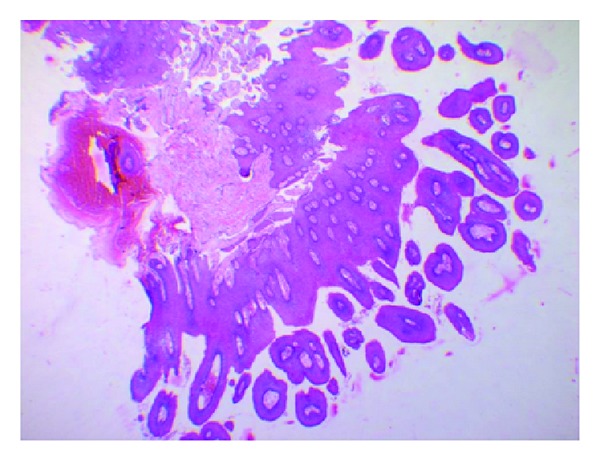
Photomicrograph of the lesion showing multiple papillary epithelial projections with fibrovascular connective tissue cores (H&E, 4x).

**Figure 8:**
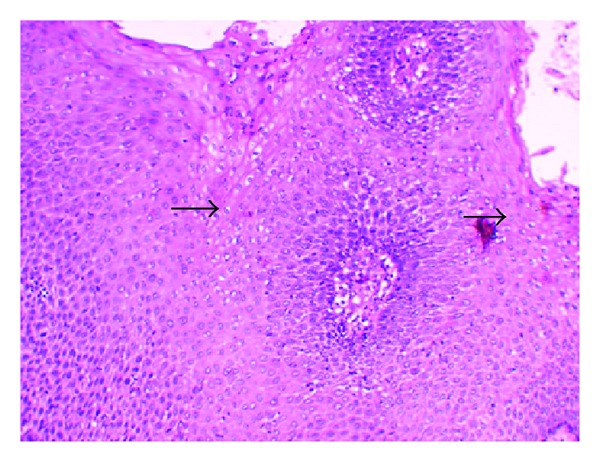
Photomicrograph of the lesion showing koilocytes (arrows) in the epithelium. Koilocytes display a hyperchromatic shrunken nucleus with clear cytoplasm (perinuclear halo) (H&E, 10x).

**Figure 9:**
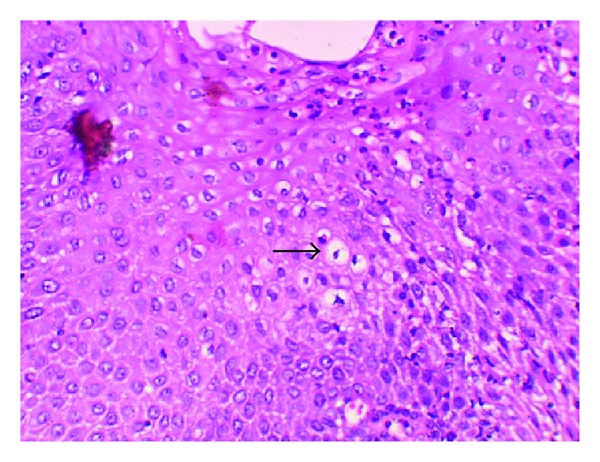
High power photomicrograph of the lesion demonstrating koilocytes (arrows) in the spinous layer (H&E, 20x).

A 65-year-old female patient presented with complaint of sensitivity of teeth since 2 months. Her past medical, dental, and personal histories were noncontributory. On extra oral examination multiple warts were seen involving face, scalp (Figures 1 and 2), and trunk. Intraoral examination showed multiple coalescent papillary projections, involving oral mucosa. The lesion was seen involving the labial gingiva of anterior teeth encroaching through the interdental papilla and involving palate (Figures 3 and 4). Predominant lesion was seen on palate extending bilaterally involving marginal gingiva of all the maxillary teeth and mid-palatine raphe and posteriorly till maxillary tuberosity region (Figure 4). Similar papillary lesions were seen involving labial gingiva of lower anterior teeth (Figure 3), dorsum of the tongue (Figure 5), and commissural areas of both right and left buccal mucosa (Figure 6). Lateral border of tongue appeared fissured with loss of papilla and melanin pigmentation. The patient observed these lesions for the past 6-7 months which were gradually spreading and were asymptomatic. Based upon the clinical features, a provisional diagnosis of HPV associated multifocal papillomatosis was established although verrucous leukoplakia, Cowden's syndrome, and verrucous carcinoma were included in the differential diagnosis. Radiological investigations revealed no underlying bony invasion. Routine blood investigations revealed no abnormality. As the present case is a disseminated form of HPV, human immunodeficiency virus (HIV) testing was done and was reported as negative. Biopsy procedures included excision of facial wart and incisional biopsy from the palate. Microscopic picture of the biopsy specimen from skin wart revealed papillomatous epidermis, evident koilocytes with shrunken nucleus surrounded by perinuclear halo in upper epidermis. Specimen from palate revealed presence of multiple papillary epithelial projections with fibrovascular connective tissue cores. The spinous layer of the epithelium demonstrated koilocytes, suggestive of papillary epithelial hyperplasia (Figures 7, 8, and 9). The palatal specimen was further subjected to molecular diagnostics using polymerase chain reaction (PCR), which revealed the presence of HPV 16 genotype using VIC HPV 16 type specific probe.

## 3. Discussion

Focal epithelial hyperplasia of the oral cavity was first reported among Navajo Indians as multiple nodular elevations, similar to those described in South American Indians and Eskimos of Greenland and Alaska [3]. The first case report was published by Dr. Heck and his team in 1965, following which it came to be known as Heck's disease. So far the literature reported less than 20 cases in humans [4]; hereby we present a rare case of multifocal epithelial hyperplasia expressing HPV 16 genotype.

Over the past few years HPV 13, 32, 1, 6, 11, and 16 were the common subtypes studied in association with the disease. Although HPV is known to be the etiological agent, possibility of genetic predisposition, nutritional deficiencies and environmental factors like poverty and lack of hygiene, role of immunosuppression have been suggested as the other risk factors [4, 5]. In the present case, genetic association was ruled out and no positive history was revealed when enquired about other risk factors; however, factors like low socioeconomic profile and poor maintenance of hygiene can possibly be attributed to the disease manifestation.

Focal epithelial hyperplasia predominantly affects patients between the 1st and 2nd decades of life with a female predilection. The most frequent site was the lower lip [4]. In this case the lesion presented at an unusual age of 65 years involving multiple sites like gingiva, palate, tongue, and commissural areas of both right and left buccal mucosa. Based upon the clinical findings, provisional diagnosis of multifocal epithelial hyperplasia was given, whereas differential diagnosis included verrucous leukoplakia and verrucous carcinoma.

The diagnosis of Heck's disease is mainly based on the clinical picture and histopathological findings. The prominent microscopic features consist of parakeratin layering, extensive acanthosis, epithelial cells of the spinous layer with enlarged nucleus, degeneration of koilocytes, and mitosoid cells [6]. Our case demonstrated multiple papillary epithelial projections with fibrovascular connective tissue cores and presence of koilocytes in the spinous layer of the epithelium favouring the diagnosis. Differential diagnosis includes condyloma acuminatum, verruca vulgaris, papilloma, irritation fibroma, and Cowden syndrome as they are known to have some clinical similarities like lesion type, color, and location.

Recent advancements in molecular biology techniques for HPV testing include light microscopy; electron microscopy; nonamplified molecular techniques like in situ hybridization, southern blot, and dot blot hybridization; target amplification using PCR; signal amplified techniques like hybrid capture technology; and gene expression using DNA microarrays [1]. In the present case the specific histopathologic features in conjunction with the clinical picture drove us towards molecular analysis, so as to rule out the possible association of human papilloma virus. The PCR analysis of the biopsy sample isolated the HPV 16 genotype; however, other common strains associated with oral lesions were ruled out using type specific probes. Studies have clearly established HPV as a definitive risk for oropharyngeal cancer and oral potentially malignant disorders [7–11]. In recent reports, HPV type 16 has been identified in 90% of HPV-associated head and neck tumours and has been found in 50% of oropharyngeal head and neck squamous cell carcinoma [1]. So far, the literature reported HPV 1, 6, 11, 13, and 32 to be commonly associated with Heck's disease and are known to be benign in nature; association of HPV 16 in our case may indicate a malignant potential of the lesion.

Lesions that do not remit or cause functional and/or aesthetic problems can be removed by various modalities like surgery, cryotherapy, electrocoagulation, laser, chemical agents (e.g., retinoic acid), or immunostimulants (e.g., interferon) [4]. Harris Ricardo et al. treated 4 cases of focal epithelial hyperplasia using 80% of trichloroacetic acid and observed resolution of the lesions within 45 days [6]. The prognosis of the disease is good, as most of the lesions remit spontaneously, although periodical clinical followups are crucial [4]. Considering the multifocal presentation of the case, we advised laser ablation of the entire lesion. Inspite of explaining about high malignant potential of the lesion, sadly the patient was not willing to receive treatment due to the asymptomatic nature of the disease. Hence, we advised periodic long-term followups to monitor the status of the lesion.

## 4. Conclusion 

The present case highlighted a rare presentation of HPV 16 infection as multifocal epithelial hyperplasia of the oral cavity with associated skin lesions. Clinicians need to be aware of such rare presentations to facilitate a prompt diagnosis and better treatment outcome, as certain strains like HPV 16 and 18 are known to have high malignant potential.
